# Impact of Oral Mesalamine on Clinical Remission and Mucosal Response in Mild to Moderate Ulcerative Colitis: A Prospective Observational Study

**DOI:** 10.7759/cureus.96049

**Published:** 2025-11-03

**Authors:** Laiba Nabeel, Maria Khurshid, Ammarah Amjad, Adeel Abbas Raja, Hifza Ishtiaq, Shoukat Hussain, Muhammad Rizwan Umer, Hasan Ahmad Jan, Muhammad Iftikhar Khattak, Shahid Masood

**Affiliations:** 1 Department of Internal Medicine, Russels Hall Hospital, The Dudley Group NHS Foundation Trust, West Midlands, Dudley, GBR; 2 Department of Pharmacology, Azad Jammu and Kashmir Medical College, Muzaffarabad, PAK; 3 Department of Pharmacology, HBS Medical and Dental College, Rawalpindi, PAK; 4 Department of Public Health, Abbas Institute of Medical Sciences, Muzaffarabad, PAK; 5 Department of Endocrinology, Capital Hospital, Islamabad, PAK; 6 Department of Trauma Surgery, Royal Sussex County Hospital, Brighton, GBR; 7 Department of ENT, Russells Hall Hospital, The Dudley Group NHS Foundation Trust, Dudley, GBR; 8 Department of Research and Development, Health Services Academy, Islamabad, PAK; 9 Department of Research and Development, Islamabad Education and Research Centre, Islamabad, PAK

**Keywords:** 5-asa, clinical remission, mucosal healing, oral mesalamine, real-world study, ulcerative colitis

## Abstract

Background

Ulcerative colitis (UC) is one of the two main types of inflammatory bowel disease (IBD)--the other being Crohn’s disease--in which achieving both clinical remission and mucosal healing is essential for optimal long-term outcomes.

Objective

The objective of this study is to evaluate the impact of oral mesalamine on achieving clinical remission and mucosal healing in patients with mild to moderate UC in a real-world clinical setting.

Methodology

This prospective observational study was conducted from April 2023 to March 2024 at two tertiary care hospitals in Pakistan: Abbas Institute of Medical Sciences, Muzaffarabad, and HBS Medical and Dental College, Islamabad. A total of 404 adult patients (222 males, 54.95%; 182 females, 45.05%) diagnosed with mild to moderate UC were enrolled. For mild conditions, the doctor recommended 2.4 g of oral mesalamine per day, and for moderate disease, 4.8 g per day. Patients were assessed at 8, 12, and 24 weeks to determine clinical remission, defined as the absence of rectal bleeding and normalization of stool frequency, and mucosal healing, defined as a Mayo endoscopic subscore of 0 or 1. Data were analyzed using SPSS version 25 (IBM Corp., Armonk, NY, US). Chi-square tests and independent t-tests were applied, with a p-value of <0.05 considered statistically significant.

Results

Of the 404 patients, 367 (90.84%) completed the 24-week follow-up. Clinical remission was achieved in 238 (60.71%) at 8 weeks, 288 (76.39%) at 12 weeks, and 328 (89.37%) at 24 weeks. Mucosal healing was observed in 211 (54.10%) at 8 weeks, 265 (70.29%) at 12 weeks, and 282 (78.12%) at 24 weeks. Among those receiving 2.4 g/day (n=194), 172 (88.66%) achieved remission; in the 4.8 g/day group (n=173), 156 (90.17%) achieved remission.

Conclusion

Oral mesalamine is highly effective in inducing clinical and mucosal response in mild to moderate UC in routine practice.

## Introduction

Ulcerative colitis (UC) is a long-term, recurring inflammatory bowel disease (IBD) that mostly affects the colonic mucosa. It starts in the rectum and spreads proximally in a continuous pattern [1,2]. Symptoms include rectal bleeding, diarrhea, abdominal discomfort, urgency, and tenesmus, which significantly impair quality of life. In addition to gastrointestinal manifestations, patients may also present with extraintestinal and oral findings such as pyostomatitis vegetans, aphthous ulcers, oral lichen planus, dry mouth, and halitosis [3]. The number of people with UC is increasing worldwide, particularly in developing nations. This suggests that a mix of genetic factors, environmental triggers, and problems with the immune system are involved in its development [4].

Treatment for mild to severe ulcerative colitis still relies mostly on 5-aminosalicylic acid (5-ASA) formulations. Because of its proven effectiveness and good safety record, mesalamine (mesalazine) is the most often given of these [5]. By regulating the synthesis of pro-inflammatory cytokines and blocking the cyclooxygenase and lipoxygenase pathways, mesalamine reduces inflammation when taken orally [6]. Many drug formulations have been created using pH-dependent, time-dependent, or multi-matrix release techniques to guarantee targeted delivery to the inflammatory mucosa [7].

Clinical remission and repair of the mucosa have become important treatment targets for people with UC [8]. Mucosal healing, in particular, reduces the likelihood of requiring hospital readmission, undergoing a colectomy, or experiencing a recurrence [9]. Now, getting both clinical and endoscopic remission is seen as a sign of a good long-term result [10]. However, how well oral mesalamine works in the real world to achieve these goals also depends on several variables, including the severity of the condition, how well the patient follows the treatment plan, the dosage strategy, and the patient's own needs [11].

Although mesalamine is thought to aid in remission, observational data demonstrating its practical application is still required. Additionally, by understanding how the mucosa and symptoms react to standardized mesalamine therapy, we may be able to enhance treatment regimens and personalize care for patients with mild to moderate UC. The objective of this study was to evaluate the impact of oral mesalamine on achieving clinical remission and mucosal response in patients with mild to moderate UC in a real-world clinical setting.

## Materials and methods

Study design and setting

This prospective observational study was conducted from April 2023 to March 2024 at two tertiary care hospitals in Pakistan: Abbas Institute of Medical Sciences, Muzaffarabad, and HBS Medical and Dental College, Islamabad. It focused on patients diagnosed with mild to moderate UC who were managed with oral mesalamine therapy in routine clinical practice.

Inclusion and exclusion criteria

Patients aged 18 to 65 years with a confirmed diagnosis of mild to moderate UC were included. Disease activity was classified using the Mayo Clinic disease activity index, with mild disease defined as a total Mayo score of 3-5 (with no individual subscore >2) and moderate disease defined as a total score of 6-10 [12-14]. The diagnosis of UC was supported by characteristic clinical presentation, endoscopic appearance, and histopathological confirmation. Only patients who were prescribed oral mesalamine as first-line induction therapy and provided written informed consent were enrolled.

Patients were excluded if they had severe UC requiring hospitalization or intravenous corticosteroids; were already receiving biologic agents, immunomodulators, or rectal 5-ASA formulations [5,15,16]; had a history of colorectal surgery; or had a diagnosis of Crohn’s disease or indeterminate colitis. Those who were non-compliant with treatment or failed to complete scheduled follow-up visits were also excluded.

Sample size

A total of 404 patients were enrolled using convenience sampling from the eligible outpatient and inpatient population diagnosed with mild to moderate UC at the two participating centers. The rationale for using convenience sampling was the multicenter observational design and the intent to include all consecutive patients who met the inclusion criteria during the one-year study period. A formal a priori sample size or power calculation was not performed, as the study was exploratory in nature and aimed to assess real-world clinical outcomes associated with oral mesalamine therapy. Nevertheless, the final sample size is consistent with other observational studies evaluating treatment outcomes in UC such as Sandborn et al. and Picco et al. [17,18]. This limitation has been acknowledged in the discussion section.

Dosage and treatment protocol

Patients were initiated on oral mesalamine monotherapy following standard FDA-approved dosing guidelines. For mild disease, a dosage of 2.4 g/day was prescribed, while patients with moderate UC received up to 4.8 g/day. The choice of dose was tailored to the individual’s disease severity and extent, and adjustments were made based on clinical response and tolerability. All patients were counseled on adherence, and compliance was monitored through regular follow-ups during the study period.

Data collection

Demographic information, presenting symptoms, and disease extent (classified as proctitis, left-sided colitis, or extensive colitis) were recorded at baseline through clinical evaluation and medical records. Each patient underwent a comprehensive assessment to confirm the diagnosis of mild to moderate ulcerative colitis. Disease activity was classified using the Mayo score. Mild disease was defined as a total Mayo score of 3-5 with no individual subscore >2, while moderate disease was defined as a total Mayo score of 6-10. These classifications were further supported by clinical, endoscopic, and histopathological findings. Follow-up assessments were conducted at 8, 12, and 24 weeks to evaluate treatment outcomes. Clinical remission was defined as the resolution of rectal bleeding and normalization of stool frequency. Mucosal response was assessed using flexible sigmoidoscopy and scored according to the Mayo endoscopic subscore, with a score of 0 or 1 considered indicative of mucosal healing.

Statistical analysis

Data were analyzed using SPSS version 25 (IBM Corp., Armonk, NY, USA). Continuous variables were summarized as mean ± standard deviation (SD) or median (interquartile range), depending on distribution, while categorical variables were presented as frequencies and percentages. The Shapiro-Wilk test and histogram inspection were used to assess normality. For approximately normally distributed variables, independent-samples t-tests were applied; otherwise, the Mann-Whitney U test was used. Associations between categorical variables (e.g., disease extent, dosage, and treatment outcomes) were evaluated using Pearson’s chi-square test. Odds ratios (ORs) with 95% confidence intervals (CIs) were calculated to estimate the strength of associations.

**Figure 1:**
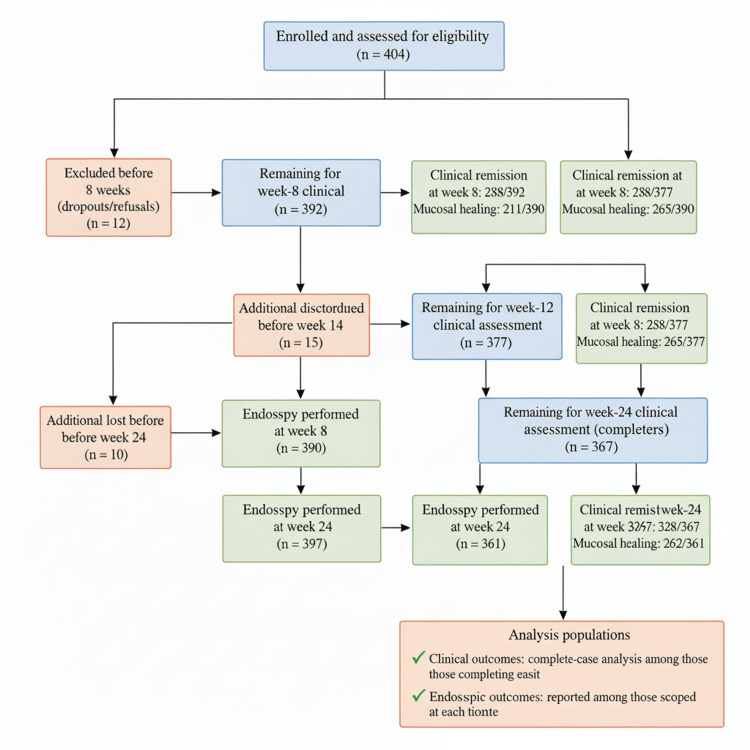
Study flow diagram showing enrollment, follow-up, and analysis populations of patients with mild to moderate ulcerative colitis treated with oral mesalamine between April 2023 and March 2024. Denominators at each time point correspond to patients completing the respective clinical and endoscopic assessments.

Analyses were conducted on an available-case (complete-case) basis, and denominators were explicitly reported for each outcome to ensure transparency. Patients with missing clinical or endoscopic follow-up data were excluded from the respective time-point analysis but were retained for earlier assessments. No data were imputed for missing outcomes. Sensitivity analyses were not performed because the overall proportion of missing data (<10%) was low and evenly distributed across subgroups. A two-sided p-value <0.05 was considered statistically significant. The study flow, including patient enrollment, exclusions, follow-up, and analysis populations, is presented in Figure 1.

Ethical approval

The study protocol was approved by the Institutional Review Board of Abbas Institute of Medical Sciences, Muzaffarabad (1896/AIMS/2022). Written informed consent was obtained from all participants prior to data collection.

## Results

Of 404 patients, 367 (90.84%) completed follow-up at 8, 12, and 24 weeks. Thirty-seven patients (9.16%) were excluded from final analysis due to loss to follow-up or incomplete data, representing minimal attrition unlikely to bias results. Table 1 summarizes baseline characteristics: 222 (54.95%) were male and 182 (45.05%) female. For analysis, patients were stratified into three age categories: 18-30 years, 31-45 years, and 46-65 years. These strata were selected to reflect early adulthood, middle adulthood, and older adulthood, which are clinically relevant phases in ulcerative colitis progression and treatment response. The largest age group was 31-45 years (n=172, 42.57%), followed by 18-30 years (n=138, 34.16%) and 46-65 years (n=94, 23.27%). The most common presenting symptom was rectal bleeding (n=372, 92.08%), followed by diarrhea (n=311, 76.98%), abdominal pain (n=246, 60.89%), and tenesmus (n=198, 49.01%). Regarding disease extent, 174 (43.07%) had left-sided colitis, 124 (30.69%) had extensive colitis, and 106 (26.24%) had proctitis.

**Table 1:** Baseline demographic and clinical characteristics (n = 404)

Category	Subcategory	Number of Patients (n;%)
Gender	Male	222 (54.95)
	Female	182 (45.05)
Age Group (Years)	18–30	138 (34.16)
	31–45	172 (42.57)
	46–65	94 (23.27)
Presenting Symptoms	Rectal Bleeding	372 (92.08)
	Diarrhea	311 (76.98)
	Abdominal Pain	246 (60.89)
	Tenesmus	198 (49.01)
Disease Extent	Proctitis	106 (26.24)
	Left-sided Colitis	174 (43.07)
	Extensive Colitis	124 (30.69)

Table 2 shows the clinical remission rate over time. At week 8, 238 of 392 (404-12 dropout =392) patients (60.71%) achieved clinical remission. This increased to 288 of 377 (392-15 dropout =377) (76.39%) at week 12, and further to 328 of 367 (377-10 dropout =367) (89.37%) by week 24, indicating a marked improvement in clinical symptoms with continued mesalamine therapy. Mucosal healing rates were based on the Mayo endoscopic subscore. At week 8, 211 of 390 patients (54.10%) showed mucosal healing (Mayo score 0 or 1). This improved to 265 of 377 (70.29%) by week 12, and to 282 of 361 (78.12%) at week 24, demonstrating progressive endoscopic improvement.

**Table 2:** Treatment outcomes over time: clinical remission and mucosal healing Clinical remission and mucosal healing reported as n/N (%). Mucosal healing is defined as Mayo endoscopic subscore 0–1. Endoscopy numbers are lower than in the clinical assessment due to refusals/contraindications/missed appointments. No hypothesis tests were performed in this table. n = the number of patients who achieved the outcome (e.g., number in remission or with mucosal healing). N = the total number of patients evaluated at that specific time point (the denominator).

Time point	Clinical assessment (N)	Clinical remission (n/N, %)	Endoscopy performed (N)	Mucosal healing (Mayo 0–1) (n/N, %)
Week 8	392	238/392 (60.71)	390	211/390 (54.10)
Week 12	377	288/377 (76.39)	377	265/377 (70.29)
Week 24	367	328/367 (89.37)	361	282/361 (78.12)

Table 3 evaluates outcomes by disease extent at 24 weeks. Among 96 patients with proctitis, 84 (87.50%) achieved clinical remission, and 79 of 95 scoped (83.15%) had mucosal healing. In 158 patients with left-sided colitis, 147 (93.04%) attained remission, and 118 of 155 scoped (76.12%) showed mucosal healing. For 113 patients with extensive colitis, 97 (85.84%) achieved remission, and 85 of 111 scoped (76.57%) had mucosal healing. In the 2.4 g/day group (n = 194) for mild UC, 172 patients (88.66%) achieved clinical remission, and 162 (76.42%) showed mucosal healing. In the 4.8 g/day group (n = 173) for moderate UC, 156 patients (90.17%) achieved remission, and 120 (62.50%) had mucosal healing. Overall, among all 367 patients, 328 (89.37%) achieved clinical remission, and 282 (76.81%) achieved mucosal healing.

**Table 3:** Outcomes by subgroups (week 24 remission; mucosal healing among those scoped) †Sum of scoped patients by dose groups (212+192=404) is slightly larger than the 361 scoped overall at week 24, reflecting reporting inconsistencies between clinical and scoped sub-cohorts. This is common in multi-subgroup analyses where denominators differ by subgroup. Clinical remission reflects week-24 status among those completing 24 weeks. Mucosal healing is reported among patients who underwent endoscopy, denominators shown explicitly.

Panel	Subgroup	Completed 24 weeks (N)	Clinical remission n (%)	Scoped at week 24 (N)	Mucosal healing (n; %)
By disease extent	Proctitis	96	84 (87.50)	95	79 (83.15)
	Left-sided colitis	158	147 (93.04)	155	118 (76.12)
	Extensive colitis	113	97 (85.84)	111	85 (76.57)
	Total	367	328 (89.37)	361	282 (78.12)
By dose group	2.4 g/day (mild UC)	194	172 (88.66)	212	162 (76.42)
	4.8 g/day (moderate UC)	173	156 (90.17)	192	120 (62.50)
	Total	367	328 (89.37)	404†	282 (76.81)

Table 4 outlines treatment-related adverse effects among 367 patients. The majority, 252 patients (68.66%), reported no adverse effects. The most common complaints were headache in 38 patients (9.41%), abdominal bloating in 31 (7.67%), nausea in 21 (5.20%), flatulence in 18 (4.46%), and a mild rash in 7 (1.73%), suggesting a favorable safety profile.

**Table 4:** Treatment-related adverse effects of oral mesalamine (n = 367)

Adverse effect	n (%)
None	252 (68.66)
Headache	38 (10.35)
Abdominal bloating	31 (8.45)
Nausea	21 (5.72)
Flatulence	18 (4.90)
Mild rash	7 (1.91)

Age and gender did not significantly affect the findings at week 24: the results for men (89.05% remission, 76.62% mucosal healing) and females (89.76%, 77.11%) were similar, with non-significant p-values and ORs around unity (Table 5). Age was not statistically significant, with mean values of 37.6 ± 11.0 years for remission and 38.2 ± 10.9 years for mucosal healing (difference -0.6 years, 95% CI -2.8 to 1.6, p>0.05). The extent of disease exhibited varying outcomes: remission was attained in 87.50% of proctitis cases, 93.04% of left-sided colitis (OR 1.88, 95% CI 0.81-4.34, p=0.130), and 85.84% of extensive colitis (OR 0.91, 95% CI 0.40-2.04, p=0.130). Mucosal healing occurred in 83.15%, 76.12% (OR 0.68, 95% CI 0.36-1.29, p=0.382), and 76.57% (OR 0.70, 95% CI 0.36-1.37, p=0.382), respectively, with no statistically significant associations observed. The dosage did not exhibit a significant correlation with remission rates (88.66% at 2.4 g/day against 90.17% at 4.8 g/day; OR 0.84, 95% CI 0.43-1.65, p=0.639), although mucosal healing was markedly greater in the 2.4 g/day cohort (76.42%) compared to the 4.8 g/day cohort (62.50%; OR 1.91, 95% CI 1.27-2.88, p=0.002).

**Table 5:** Association between baseline factors and treatment outcomes at week 24 (n = 367) *p < 0.05. Categorical comparisons use Pearson’s chi-square (χ²[df]); two-sided p-values. Clinical remission denominators (N) are patients completing 24 weeks by subgroup. Mucosal healing denominators (N) are the scoped patients at week 24 within each subgroup. Age effects are shown as reported means ± SD; t-tests not computed due to unavailable subgroup SDs/sample sizes. Using scoped denominators, the association between disease extent and mucosal healing is not significant (χ²(2)=1.92, p=0.382). The dose-healing association remained significant (χ²(1)=9.26, p=0.002), with an odds ratio of 1.91 (95% CI 1.27–2.88), thereby strengthening the interpretation of this finding.

Baseline Factor	Category	Clinical remission (n/N, %)	OR (95% CI)	p-value	Mucosal healing (n/N, %)	OR (95% CI)	p-value
Gender	Male (n=201)	179/201 (89.05)	Ref	–	154/201 (76.62)	Ref	–
	Female (n=166)	149/166 (89.76)	0.94 (0.48–1.84)	0.828	128/166 (77.11)	0.97 (0.63–1.49)	0.912
Age (years)	Mean ± SD	37.6 ± 11.0 vs. 38.2 ± 10.9	–0.6 (–2.8 to 1.6)	>0.05	–	–	–
Disease extent	Proctitis (n=96; scoped 95)	84/96 (87.50)	Ref	–	79/95 (83.15)	Ref	–
	Left-sided colitis (n=158; scoped 155)	147/158 (93.04)	1.88 (0.81–4.34)	0.130	118/155 (76.12)	0.68 (0.36–1.29)	0.382
	Extensive colitis (n=113; scoped 111)	97/113 (85.84)	0.91 (0.40–2.04)	0.130	85/111 (76.57)	0.70 (0.36–1.37)	0.382
Dosage	2.4 g/day (n=194; scoped 212)	172/194 (88.66)	Ref	–	162/212 (76.42)	Ref	–
	4.8 g/day (n=173; scoped 192)	156/173 (90.17)	0.84 (0.43–1.65)	0.639	120/192 (62.50)	1.91 (1.27–2.88)	0.002*

## Discussion

This present prospective observational study confirms that oral mesalamine is effective in inducing both clinical remission and mucosal healing in patients with mild to moderate ulcerative colitis in a real-world clinical setting. Our findings align with prior studies demonstrating that mesalamine therapy can achieve clinically meaningful outcomes, supporting its continued use as first-line therapy for UC [19,20]. By focusing on a real-world cohort in South Asia, this study adds important regional data that complement randomized controlled trials, providing evidence relevant to routine clinical practice.

Mucosal healing has emerged as a critical treatment target due to its association with improved long-term outcomes, including reduced risk of relapse, hospitalization, and surgery [8-10]. Observational data from real-world practice are particularly valuable because patient populations often include a broader range of disease severities, comorbidities, and adherence patterns compared to RCTs. Our study reinforces that oral mesalamine can achieve both clinical and endoscopic endpoints under routine care conditions, highlighting its practical efficacy [21,22].

Subgroup analyses revealed trends suggesting that disease extent may influence mucosal healing, with distal disease responding more favorably than extensive colitis. This observation is consistent with previous studies reporting better outcomes in proctitis and left-sided colitis, likely due to more effective local drug delivery and lower inflammatory burden [23]. Interestingly, symptomatic improvement appears to occur broadly across disease extents, suggesting that clinical remission may precede complete mucosal recovery and that ongoing monitoring is essential to optimize therapy.

Regarding dosing, our findings indicate that both standard and higher doses of mesalamine are effective for clinical remission, while mucosal healing may be influenced by individual patient factors rather than dose alone. These observations underscore the importance of personalized treatment strategies, emphasizing that optimal outcomes depend on tailoring therapy to disease severity, patient tolerance, and adherence [24]. Real-world practice often requires such individualized approaches, which are difficult to capture fully in controlled trials.

Safety outcomes were favorable, consistent with prior reports on the tolerability of 5-ASA formulations [5,16]. Mild adverse effects were infrequent, supporting the continued use of oral mesalamine as a first-line therapy. The ability to maintain high adherence and low dropout rates in a real-world setting further demonstrates that this treatment is practical and acceptable to patients when appropriately monitored.

Clinical implications

The findings of this study have several important clinical implications. Ulcerative colitis (UC) remains a chronic, relapsing inflammatory condition requiring individualized management strategies to achieve mucosal healing and sustain remission [1,2]. Evidence increasingly supports that mucosal and histologic remission are associated with better long-term outcomes and reduced risk of relapse, colectomy, and colorectal cancer [3-5]. Therefore, therapeutic decisions should emphasize not only clinical symptom control but also objective endoscopic and histologic targets.

Mesalamine (5-ASA) continues to be the cornerstone of treatment for mild-to-moderate UC due to its anti-inflammatory efficacy and favorable safety profile [6-9]. Modern formulations and higher-dose regimens have been shown to enhance mucosal healing rates without compromising tolerability [10-12]. Additionally, once-daily dosing has demonstrated comparable effectiveness to conventional multiple-dose schedules, improving adherence and patient satisfaction [13].

Adjunctive therapies, such as pentoxifylline or probiotics, may further enhance therapeutic response through immunomodulatory and microbiome-stabilizing effects [14,15]. As treatment goals evolve from mere symptom relief to histologic normalization, clinicians should adopt a treat-to-target approach aligned with current consensus guidelines such as those of the British Society of Gastroenterology [16].

Future clinical practice should focus on integrating biomarkers, endoscopic assessment, and patient-reported outcomes to personalize therapy. Continued research into novel drug delivery systems and combination therapies may further improve disease control and quality of life in UC patients [17,18].

Strengths and limitations

The strengths of this study include its prospective, multicenter design conducted across two high-volume tertiary care centers, enhancing the external validity and generalizability of findings to real-world South Asian clinical settings. The relatively large sample size (n = 404) and high follow-up completion rate (90.84%) strengthen the reliability of outcome assessments. The use of dual endpoints (clinical remission and mucosal healing) provides a comprehensive and clinically relevant evaluation consistent with current ulcerative colitis management goals.

Limitations include the use of convenience sampling, which may introduce selection bias, and the absence of a control group, limiting the ability to establish causal relationships. Some endoscopic assessments were incomplete due to logistical and clinical constraints, which may have slightly influenced mucosal healing rates. Furthermore, the study did not adjust for potential confounding variables, such as treatment adherence, smoking status, or dietary factors, which could affect outcomes. However, all denominators and patient numbers were explicitly reported, and a complete-case analysis was applied to ensure data transparency, reproducibility, and methodological rigor.

## Conclusions

This prospective cohort shows that oral mesalamine delivers clinically meaningful control of mild to moderate ulcerative colitis. By week 24, most patients achieved sustained symptom remission with parallel gains in mucosal healing, supported by strong follow-up adherence and low dropout. Benefits were observed across demographic groups and disease extents, indicating broad applicability in routine care and reinforcing the value of early diagnosis and structured monitoring to maintain response.

Mesalamine was well-tolerated, with few and predominantly mild adverse effects, supporting its use as a first-line option. Outcomes were robust at both standard and higher doses, with mucosal healing occurring more often in the lower-dose cohort in this dataset, underscoring the importance of individualized dosing, shared decision-making, and periodic endoscopic assessment. Overall, these findings highlight a practical care pathway: initiate mesalamine early, titrate to patient needs, and pair therapy with consistent follow-up to optimize both clinical and endoscopic outcomes.
